# Effects of Vacuum Frying on the Preparation of Ready-to-Heat Batter-Fried and Sauced Chub Mackerel (*Scomber japonicus*)

**DOI:** 10.3390/foods10081962

**Published:** 2021-08-23

**Authors:** Gabriel Tirtawijaya, Mi-Jeong Lee, Bertoka Fajar Surya Perwira Negara, Woo-Hee Cho, Jae-Hak Sohn, Jin-Soo Kim, Jae-Suk Choi

**Affiliations:** 1 Seafood Research Center, IACF, Silla University, 606, Advanced Seafood Processing Complex, Wonyang-ro, Amnam-dong, Seo-gu, Busan 49277, Korea; tirtawijayag@yahoo.com (G.T.); ftrnd10@silla.ac.kr (M.-J.L.); ftrnd12@silla.ac.kr (B.F.S.P.N.); ftrnd3@silla.ac.kr (W.-H.C.); jhsohn@silla.ac.kr (J.-H.S.); 2 Department of Food Biotechnology, College of Medical and Life Sciences, Silla University, 140, Baegyang-daero 700 beon-gil, Sasang-gu, Busan 46958, Korea; 3 Department of Seafood and Aquaculture Science, Gyeongsang National University, 38 Cheongdaegukchi-gil, Gyeongsangnam-do, Tongyeong-si 53064, Korea

**Keywords:** chub mackerel, nutritional quality, ready-to-heat, shelf life, vacuum frying

## Abstract

Chub mackerel (CM) is a commercial fish in Korea, owing to its availability and nutritional values. This study aimed to develop a ready-to-heat (RTH) Korean preparation of CM, known as *Godeungo gangjeong.* We utilized vacuum frying technology to fry the CM and evaluated its quality. Conventional frying with a deep fryer was performed in parallel to assess the superiority of the vacuum fryer. We optimized the frying conditions of vacuum frying (VBF) and deep frying (DBF) using response surface methodology. At optimum conditions of 95 °C for 7 min 42 s, VBF produced better sensory, chemical, and microbial properties than DBF at 190 °C for 5 min 30 s. The nutritional values, including amino acid and fatty acid contents, were investigated and found to be higher in VBF than in DBF. Sensory properties also showed better scores on VBF than DBF, especially in appearance, aroma, taste, and overall acceptability. The VBF produced lower volatile basic nitrogen (VBN), thiobarbituric acid reactive substances (TBARS), and total bacterial count (TBC) than DBF. The findings confirmed that vacuum frying is a better option to produce RTH *Godeungo gangjeong*, since it provides less oxidation and maintains the product quality. Using the Arrhenius approach, the product was concluded to preserve both quality and safety for 9 months of storage at −18 °C.

## 1. Introduction

The chub mackerel (CM; *Scomber japonicus*) is a pelagic fish that is distributed in subtropical waters. The fish swims around the East Sea (Sea of Japan) and the Yellow Sea (East China Sea); fishing grounds are located around the East Sea and South Sea of Korea [1]. In 2020, the Korean Ministry of Oceans and Fisheries reported that CM was the second-largest caught fish after anchovy, with a catch as high as 77,000 tons. Thus, CM is one of the most important commercial fish in Korea.

CM is a source of valuable nutrition, such as proteins, lipids, and minerals, to humans [2,3]. It can be processed by various methods, including smoking, grilling, braising, canning, and frying [4,5,6,7]. Fried CM is a popular dish; in Korea, batter-fried CM is mixed with a special sauce to prepare *Godeungo gangjeong*. The latter is prepared by coating CM with batter, followed by frying and then mixing with a sauce. This food is exclusively offered in restaurants or prepared at home; no commercial products, such as ready meals, have been developed yet. Currently, the deep-frying method is utilized in the preparation of *Godeungo gangjeong*.

The deep-frying method involves immersion of the food in hot oil at temperatures of 150–200 °C, and the temperature is influenced by the food composition and heat-mass transfer properties [8]. The high temperature and oxygen contact in the deep-frying method increases the thermo-oxidative degradation of foods [9]. Deep-fried sturgeon (*Huso huso*) had previously been reported to have altered fatty acid composition with decreased contents of eicosapentaenoic acid (EPA) and docosahexaenoic acid (DHA) [10]. Furthermore, Chaula et al. [11] had shown that the decrease in polyunsaturated fatty acids (PUFA) in deep-fried sardine was due to oxidative damage during high-temperature processing.

In order to develop a ready-to-heat meal, we focused on products that could provide both nutrition and convenience. Vacuum frying is a method that involves lower pressure, lower temperature, and lesser oxygen content than deep frying; it preserves the nutrition of foods and maintains their natural flavor and color [12,13]. Vacuum frying of fish patties showed less oxidation compared to conventional frying [14]. Foods processed by vacuum frying are also reported to have brighter color [15], and lower hardness and acrylamide content [16], and require shorter time and lower temperature [17] than deep-fried foods. Nurdiani et al. [18] showed that vacuum frying of catfish contained less water and oil than conventional frying, which might eventually increase its shelf life.

The current study aimed to determine the optimal vacuum frying conditions and their influence on the nutritional composition of sauced CM. The sensory, chemical, and microbial properties in the deep-frying method were also analyzed and compared with those in the vacuum frying method. Sauced CM samples after various storage periods were evaluated to estimate their shelf life as a ready-to-heat meal.

## 2. Materials and Methods

### 2.1. Preparation of Mackerel Sample

Frozen mackerel (*S. japonicus*) was provided by EBADA Fishery Co., Ltd., Busan, Korea. The mackerel samples were thawed using a high-frequency defroster (TEMPERTRON FRT-10, Yamamoto Vinita Co. Ltd., Osaka, Japan) for 20 min. They were subsequently deboned, cleaned, and cut into a size of 3.5 × 5.0 cm (width × length) with an approximate weight of 25.0–25.5 g.

### 2.2. Preparation of Batter

The batter formulation included frying powder mix and water at a ratio of 1/1.55 (*w/v*). The flour mix consisted of soft flour (70%), rice flour (8%), corn starch (15%), roasted rice flour (1.55%), refined salt (1.5%), garlic powder (0.75%), ground pepper (0.4%), baking powder (1.35%), and sugar (1.45%). The ingredients of the frying powder mix were purchased from a local market in Busan, Korea.

### 2.3. Optimization of Frying Conditions

Pieces of mackerel were dipped in batter and fried in canola oil (Cargill Inc., Camrose, Canada). Two frying methods, vacuum frying and deep frying, were used. Samples were deep-fried in an electric fryer WS-EFS20 (Woosung Enterprise Co., Ltd., Seoul, Korea) and vacuum fried in a vacuum fryer BT-1E (Kiyomoto Co., Ltd., Nobeoka, Japan). The frying conditions of each method were optimized based on the design of the experiment using response surface methodology (RSM). The experimental design was generated based on two factors (temperature and time), three center points, and a five-level central composite design. A total of 11 runs of the experiment, consisting of low, central, and high factor levels, were used to evaluate the sensory properties (overall acceptance; OA), volatile basic nitrogen (VBN) content, and thiobarbituric acid reactive substances (TBARS) content. The conditions of both vacuum and deep-frying methods are listed in Table 2.

**Figure 1:**
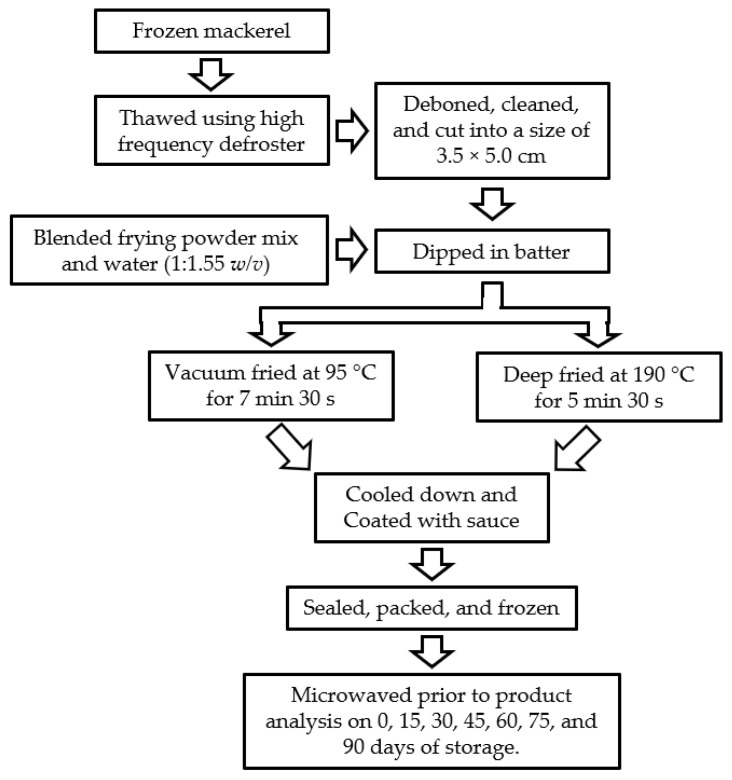
Processing steps of batter-fried chub mackerel with sauce.

Fried mackerel samples were cooled and coated with sauce; the composition of the sauce is listed in Table 1. Sauced mackerel samples were packed in a polypropylene plastic bowl (Ø, 10.0 cm; New Ecopack Co., Ltd., Jeonju, Korea) prior to sealing with a TPS-TS3T packaging machine (TPS Co., Ltd., Hwaseong, Korea) at 180 °C for 5 s. Processing steps of the fried and sauced mackerel are shown in Figure 1. A total of 25 packages for each time and temperature of storage was prepared. Samples were frozen at −18 °C prior to analysis of sensory, chemical, and microbial properties, and nutritional composition. Samples for shelf life analysis were frozen at three different temperatures (−13, −18, and −23 °C) as described in Section 2.8.

### 2.4. Analysis of Sensory Properties

The appearance, aroma, taste, texture, and OA were evaluated to compare the sauced mackerel obtained using different frying methods. Sensory analyzes for comparison of VBF, DBF, VBFS, and DBFS samples previously stored at freezer −18 °C. Each different sample treatment was coded before being served to the panelists. Panelists evaluated the samples based on a randomized list produced with a random sampling table generated using Microsoft Excel 2010. Samples for shelf life evaluation were frozen at three different temperatures as described in Section 2.8. Frozen sauced mackerel samples were microwaved (RE-M50, Samsung Electronics Co. Ltd., Seoul, Korea) for 1.5 min at 700 W before analyzing its sensory properties. The temperature of samples served was 50–55 °C. The evaluation was performed by 21 trained panelists (age range, 25–40 years) using a hedonic scale of 1 (dislike extremely), 2 (dislike very much), 3 (dislike moderately), 4 (dislike slightly), 5 (neither like nor dislike), 6 (like slightly), 7 (like moderately), 8 (like very much), and 9 (like extremely). A scale of 5 was used as the threshold, such that sensory properties below that would be considered unacceptable [19]. The sensory analysis session started at 10 a.m. for a maximum of 2 h in a designated room with a temperature of 23 ± 2 °C. The samples were served on the table wrapped in white paper. Each table was equipped with mineral water, a cup, chopsticks, a spoon, and plain biscuits. The distance between the panelist was 1 m. The 21 panelists in this study were the same until the end of the study. The sensory properties were analyzed with the approval of the institutional review board of Silla University (Busan, Korea).

### 2.5. Analysis of Chemical Properties

Chemical properties of the processed mackerel were analyzed next, including the thiobarbituric acid reactive substances (TBARS) and volatile basic nitrogen (VBN) content. The chemical properties were measured simultaneously with the sensory properties, as described in a previous study [20]. The measurement of VBN values was prepared by homogenizing 5 g of a sample and 25 mL of distilled water (DW) using the homogenizer WiseTis SHG-15D (SciLab Co. Ltd., Seoul, Korea). The homogenized sample was centrifuged and filtered to separate the supernatant. The supernatant was analyzed using Conway micro-diffusion. The measurement of TBARS values was prepared by homogenizing 5 g of a sample and 12.5 mL of trichloroacetic acid (20%) in phosphoric acid (2 M). After homogenization, DW was added to a total volume of 25 mL. The sample was centrifuged at 4 °C for 15 min to separate supernatant. The 2 mL of supernatant was mixed with 2 mL of thiobarbituric acid (TBA 0.005 M). The mixture was incubated in a water bath at 95 °C for 30 min. The absorbance of the mixture was measured at wavelength 530 nm using Spectrostar nano (BMG Labtech Ltd., Ortenberg, Germany). The measurement of VBN and TBARS were performed in triplicate.

### 2.6. Analysis of Microbial Properties

The microbial properties analyzed in the processed mackerel included the total bacterial count (TBC), *Staphylococcus aureus*, *Salmonella* spp., and *Escherichia coli* colonies. Analysis was conducted as described in a previous study [20] and represented as log CFU/g. Briefly, 10 g of sample was diluted with sterile 0.9% saline solution in a sterile plastic bag. The sample was homogenized using Bagmixer (Interscience Co., Ltd., Osaka, Japan). Diluted samples were spread on specific Petrifilm plates (3M Korea Ltd., Seoul, Korea) for TBC, *S. aureus*, *Salmonella* spp., and *Escherichia coli* in triplicate. Plates were incubated at 35 °C for 2 days. The number of microbial was counted following the manufacturer’s instructions.

### 2.7. Analysis of Nutritional Composition

The nutritional composition of the processed mackerel was analyzed following the method of the Association of Official Analytical Chemists [21]. The fatty acid and amino acid profiles were obtained from standard chemicals based on the method described in a previous study [5]. The fatty acid contents of raw and processed CM were calculated to obtain the lipid quality indices, including hypocholesterolemic/hypercholesterolemic (h/H), atherogenic (AI), and thrombogenic (TI) indices, which were in turn calculated as described by Czech et al. [22].

(1) $$h/H=\frac{C18:1+\;C18:2+\;C18:3+\;C20:3+\;C20:4+\;C20:5+\;C22:4+\;C22:5+\;C22:6}{C14:0+\;C16:0}$$

(2) $$\mathrm{AI}=\frac{\left(4\times C14:0\right)\;+\;C16:0}{\left(\omega 6\right)\;+\;\left(\omega 3\right)\;+\mathrm{MUFA}}$$

(3) $$\mathrm{TI}=\frac{\left(C14:0+\;C16:0+\;C18:0\right)}{\left(0.5\times \;\mathrm{MUFA}\right)\;+\;\left(0.5\times \omega 6\right)\;+\;\left(3\times \omega 3\right)\;+\;\left(\frac{\omega 3}{\omega 6}\right)\;}$$

where C18:0 to C22:6 = carbon chain of the fatty acids, ω6 = omega 6 polyunsaturated fatty acids, ω3 = omega 3 polyunsaturated fatty acids, and MUFA = monounsaturated fatty acids

### 2.8. Prediction of Shelf Life

The shelf life of the processed mackerel was predicted according to guidelines from the Ministry of Food and Drug Safety (MFDS), Korea. Vacuum batter-fried and sauced CM samples were stored at temperatures of −13 °C, −18 °C, and −23 °C, and analyzed every 15 days for a total of 75 days. The data of overall acceptance, VBN, TBARS, and TBC were used for shelf life prediction using the Arrhenius kinetic theory. The rejection criteria for OA, VBN, TBARS, and TBC were set at score 5, 25 mg%, 5 mg MDA/kg, and 5 log CFU/g, respectively. The final shelf-life prediction was made by multiplying the Arrhenius calculation result with a safety factor of 0.8 [23].

### 2.9. Statistical Analysis

The design of experiment (DOE), analysis of response surface and response optimizer, analysis of variance (ANOVA), and Tukey’s post hoc multiple comparison tests (*p* < 0.05) were performed using Minitab ver. 19.0 (Minitab LLC, State College, PA, USA). The shelf life prediction was analyzed using the Visual Shelf Life Simulator for Foods provided by MFDS (https://www.foodsafetykorea.go.kr (accessed on 10 June 2021)). The analysis of microbial and chemical properties was done in triplicate.

## 3. Results and Discussion

### 3.1. Optimum Conditions of Vacuum and Deep Frying Treatments

Optimization of vacuum and deep-frying conditions was performed using RSM. Battered CM samples were treated according to DOE and analyzed for the dependent varia-bles, namely OA, VBN, and TBARS. The DOE of vacuum and deep frying (Table 2) differed in the independent variables based on our preliminary experiment. Temperature (X_1_) and time (X_2_) were the independent variables. The OA scores represented the sensory properties of batter-fried CM. The freshness and lipid oxidation of batter-fried CM were described by VBN and TBARS values.

In this study, vacuum frying used a lower temperature and longer time than deep frying. Vacuum batter-fried CM (VBF) showed an OA score of 4.02–8.70, VBN values of 10.53–12.29 mg%, and TBARS values of 1.27–1.74 mg MDA/kg. The overall acceptance score and VBN and TBARS values of deep batter-fried CM (DBF) were 6.60–8.38, 11.94–14.39 mg%, and 1.31–1.76 mg MDA/kg, respectively. The low OA score (4.02–4.13) in the VBF sample was due to its flesh remaining uncooked at the frying temperatures of 88–90 °C. At a frying temperature of 95 °C and a duration of 7 min, VBF achieved the highest OA score among all treatments, including all DBF samples. In DBF samples, the high temperature (200 °C) and long time (6.5 min) of the process resulted in a low OA score (6.6) with a dark brown color. VBN and TBARS values did not differ significantly with temperature and time of frying in both VBF and DBF samples, but they did tend towards higher values in the DBF samples than in the VBF samples.

Models from the RSM analysis showed significance in OA (*p* < 0.05) but not in VBN and TBARS (Table 3). Therefore, the response optimizer was only applied to the OA data. The predictive model built for the corresponding analysis is presented in Table 4. For VBF, the predictive model was fully quadratic, while for DBF, it was linear quadratic since its two-way interaction was not significant. The *R*^2^ values of VBF and DBF were 97.00% and 80.55%, respectively. The values explained the coefficient of determination, describing the closeness of data to the built predictive models. The lack of fit for both VBF and DBF was not significant (*p* > 0.05), indicating that the predictive models were accurate in predicting the optimal temperature and duration for batter-fried CM.

**Figure 2:**
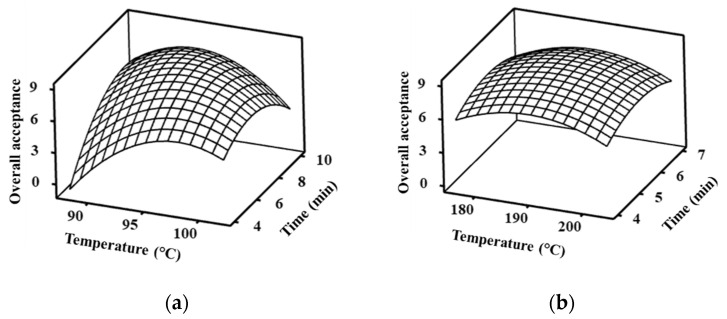
The response surface graph for overall acceptance (OA) of (**a**) vacuum batter-fried and (**b**) deep batter-fried chub mackerel at the designated temperature and time.

The predictive model in Table 4 shows the predictive values of OA response, which were plotted into a three-dimensional graph (Figure 2). The OA scores of VBF (Figure 2a) increased with increasing temperature and time but decreased after an optimal temperature of 95.21 °C and time of 7.70 min. In DBF samples, OA scores showed the same pattern as the VBF samples. The optimal temperature and time of the DBF sample were 189.58 °C and 5.54 min, respectively. The study was continued with frying conditions adjusted to the configuration settings of the fryer, as follows: 95 °C for 7 min 42 s (VBF) and 190 °C for 5 min 30 s (DBF).

### 3.2. Sensory Properties of Processed Chub Mackerel

The sensory properties of processed chub mackerel were evaluated to compare the effects of different frying treatments and the addition of sauce to CM, and the results are presented in Table 5. Sensory scores of all the samples were above the “like very much” category. All sensory property scores of the VBF samples were higher than those of the DBF samples. Appearance, taste, and OA were significantly different between the VBF and DBF treatments (*p* < 0.05). The DBF samples had a darker brown color and stronger frying odor than the VBF samples; therefore, the appearance, aroma, and taste scores of DBF samples were lower than those of VBF samples. The appearance and texture of fish have a considerable impact on customer acceptance [24]. On the contrary, Negara et al. [25] reported that vacuum fried CM had a lower sensory score than deep fried. This current study confirms that batter treatment on CM enhanced consumer acceptability. Vongsawasdi et al. [26] found that fried batter chicken decreased the oil absorption and moisture loss, thereby improved sensory properties.

The vacuum condition in a vacuum fryer can preserve the natural taste and color due to the reduction of food oxidation [27,28]. The addition of sauce in VBF and DBF samples improved their sensory properties further, and the appearance, in particular, achieved a “like extremely” score. Aroma, taste, and OA scores of the DBF samples increased significantly with the addition of sauce. VBFS samples showed the highest sensory properties among the four treatments and hence were considered to be the most preferred by panelists. The results, together, suggested that frying battered CM in a vacuum fryer produced better sensory properties with improved appearance, aroma, texture, taste, and OA. The addition of sauce increased the shimmering red color and distinctive seasoning taste. The addition of sauce to the DBF sample resulted in a significant increase in aroma, taste, and OA (*p* < 0.05). This might be owing to the sauce’s strong flavor and the presence of aromatic spices (Table 1). Thus, although the VBFS sample had the highest sensory properties score, it was not significantly different from the DBFS sample.

### 3.3. Chemical and Microbial Properties of Raw and Processed Chub Mackerel

The chemical and microbial properties of processed CM were compared with those of raw CM. The chemical properties (VBN and TBARS contents) of raw CM, as the initial conditions, were lower than those of the processed CM (Table 6). During frying, several physicochemical properties change, such as browning, crust formation, and protein degradation [29]. Degradation of proteins in fried foods results in changes in the VBN values. The latter were significantly increased by 1.52–1.58 times than in raw CM (*p* < 0.05) in this study. While the use of a vacuum fryer increased the VBN value, it was lower than with a deep fryer. The VBN value is commonly used to determine the freshness of fish. The formation of VBN is related to the breakdown of proteins by microbial and enzymatic activities [30]. Low-molecular-weight compounds produced from protein breakdown, such as amines, aldehydes, ketones, and esters, are constituents of basic nitrogenous compounds in VBN [31]. Several compounds in seafood, including ammonia, dimethylamine, and trimethylamine, along with other volatile basic nitrogenous compounds, contribute to the total VBN [32]. In this study, frying treatments caused thermal degradation of proteins that possibly increased the VBN values. Jiang et al. [33] had reported that the proteins of bighead carp were degraded into low-molecular-weight compounds during heating treatments at 90–120 °C. Moreover, several amino acids produced ammonia during thermal treatment at 180 °C [34]. According to Domínguez et al. [35], cooking temperature and time significantly generated volatile compounds in meat. Higher temperatures in deep frying, compared to those in vacuum frying, might be the reason for the corresponding higher VBN values. As a result, the application of vacuum frying produced a lower VBN value in batter-fried.

TBARS values represent lipid oxidation in foods. Like the VBN values, the TBARS values were increased significantly by 1.31–1.46 times (*p* < 0.05) due to the frying process, with a lower value obtained in vacuum-fried samples than in deep-fried samples. The increase in TBARS values indicated an increase in malondialdehyde amounts (MDA) caused by the oxidation of fatty acids, hence producing rancidity [36,37]. Several studies have shown that frying, roasting, baking, and boiling fish increase the MDA values [38,39,40]. Vacuum frying of fish patties showed less oxidation compared to the conventional frying method [14]; the presence of oxygen and high temperatures beyond 150 °C is known to cause lipid oxidation [7]. Vacuum-frying of battered CM produced a lower TBARS value than deep frying since the former minimized oxygen exposure to the fried CM and used frying temperatures lower than 100 °C.

The total bacterial count (TBC) for *Salmonella*, *Staphylococcus*, and coliform groups was considered for the microbial properties; none of the samples showed any pathogenic microbe (*Salmonella*, *Staphylococcus*, and coliform groups). The results indicated that the raw CM used in this study had been treated hygienically. The TBC values of processed CM were significantly lower than those of raw CM (*p* < 0.05). The vacuum frying method showed a similar TBC as the deep-frying method, hence confirming that both frying methods were effective in reducing TBC in the processed CM. Thermal treatments are commonly used in fish processing to inactivate undesired microorganisms, thereby producing safe products and extending their shelf life [41].

The addition of sauce in both vacuum and deep batter-fried CM decreased the VBN (5.32–6.78%), TBARS (4.48–7.38%), and TBC (19.48–22.74%) values. The reduction of these values could be due to the sauce causing dilution of the sample. The acceptable limits of VBN, TBARS, and TBC have been reported to be 25 mg%, 5 mg MDA/kg, and 5 log CFU/g, respectively [42,43]. Overall, the chemical and microbial properties of all treated samples met the recommended values for good and safe food for human consumption.

### 3.4. Nutritional Composition of Raw and Processed Chub Mackerel

Fish is a rich source of good lipids, proteins, and micronutrients for a healthy human diet. The nutritional composition of raw and processed CM is shown in Table 7. In general, the macro- and micro-nutrient contents of batter-fried CM (VBF and DBF) were higher than those of raw CM. A possible reason for this increase is the reduction in moisture content. The lowest moisture content was found in the VBF samples (24.02%), while it was 39.54% and 69.36% in DBF and raw samples, respectively. The results are consistent with those of a previous study by Moon et al. [44], which revealed that cooking CM by frying or using an oven or microwave oven increased protein, lipid, and ash and decreased the moisture content. The lipid and protein contents of Cameroonian mackerel (*Scomber scombrus*) and Indian mackerel (*Rastrelliger kanagurta*) also increased after frying [45,46]. Rahman et al. [46] had reported that the increase in lipid content of fried fish fillet was mainly due to the absorption of oil and leaching out of the water from the fish during deep frying. The moisture content of fried breaded shrimp [16] and fried gilthead sea bream [47] was decreased by vacuum frying but lower than atmospheric frying. Andr és-Bello et al. [47] found that the moisture loss in vacuum fried gilthead sea bream was significantly affected by frying time but not by frying temperature. Thus, we assume that VBF has lower moisture content than DBF as a result of the longer frying time. However, this condition was in conformity with consumer preferences based on analysis of the sensory properties. The moisture contents of VBFS and DBFS samples were increased by 16.9% and 13.9%, respectively, due to the addition of sauce. The sauce contained water from its ingredients, such as corn syrup, tomato ketchup, chopped garlic, soy sauce, apple juice, oyster sauce, vinegar, cooking wine, lemon juice, and purified water. The increase in the moisture content of VBFS and DBFS was improved the texture property, although not significantly.

The VBF sample showed higher protein and good lipid contents, such as DHA and EPA, compared to the DBF samples and the samples with sauce. Results suggested that the use of a vacuum fryer would provide better nutritional content than the use of a deep fryer. Oduro et al. [7] had reported that the increase in lipid content of fried fish was related to the absorption of vegetable oil during frying. A lower temperature in the vacuum fryer (than in the deep fryer) might also pre-serve the proteins and lipids of batter-fried CM. The increase in temperature and time during heat processing increases both protein denaturation and loss of vitamins and minerals [24]. Denaturation of proteins at high temperatures causes damage to cell structures and hardens the food [48].

The amino acids of raw and processed CM were evaluated and shown in Table 8. Since amino acids are the building blocks of proteins, changes in amino acids showed a similar trend as changes in protein content. The major amino acids in raw CM were glutamic acid, aspartic acid, lysine, and leucine; the result was similar to that observed by Oduro et al. [7]. Threonine, valine, isoleucine, leucine, phenylalanine, lysine, methionine, tryptophan, histidine, and arginine are reported to be essential amino acids for humans [49].

All the amino acids were higher in amount in batter-fried CM than in raw CM. The VBF sample showed the highest amino acid content, with the majority being glutamic acid, aspartic acid, lysine, and leucine. The total amino acid content of the VBF sample was 6.4-fold higher than that of the raw sample, whereas the total amino acid content of the DBF sample was only 2.7-fold higher than that of the raw sample. The lower increase in the amino acid content of batter-fried fish in the deep-frying method indicated degradation of amino acids due to high temperatures. Frying in a deep fryer decreased the quality of amino acids in CM compared to frying in a vacuum fryer. Oluwaniyi et al. [50] had reported that deep frying at temperatures 175–200 °C for 15 min significantly decreased the total amino acid content of fish. The major loss of essential amino acids occurred for lysine, which could be due to the Maillard reaction during the deep frying of mackerel [51]. The stability of amino acids had been previously reported by Sohn and Ho [32]; they found aspartic acid to be stable at 110–150 °C and degraded at temperatures above 150 °C. Moreover, the degradation of cysteine and asparagine gradually increased in the range of 110–180 °C, and glutamine showed high degradation at 110 °C.

Fatty acid compositions of the raw and processed CM were evaluated next. Table 9 shows the lipid profile and indices of the raw and processed CM. The total SFA of processed CM decreased 25.05–54.90%, with the lowest SFA obtained from the VBF sample (15.32%). The total MUFA of raw CM was 41.71%, which increased to 52.91% and 47.55% for the VBF and VBFS samples, respectively. A similar trend was reported by Gaurat et al. [52], where the SFA of fried catla fish decreased, while its MUFA increased. The frying process affects the lipid uptake from cooking oil and the removal of water [53]. Pan et al. [16] reported that the increase in oil absorption is related to the increasing moisture loss and frying time of fried breaded shrimps. They showed vacuum frying produced lower moisture loss, which resulted in lower oil absorption than atmospheric frying. In contrast with the current study, the moisture loss was higher in vacuum frying than deep frying. This might be related to the higher frying time in vacuum frying (7 min 42 s) than deep frying (5 min 30 s). Increased moisture loss could be the reason for the increased lipid content in the sample. Canola oil, used in this study as the cooking oil, is rich in MUFA (63.3%) content and low in SFA (7.4%) content [54]. Therefore, the increase in MUFA content of VBF and DBF samples was possibly caused by lipid uptake from cooking oil. The fatty acid content, which is high and increased after frying, was oleic acid and linoleic acid. These conditions are caused by the use of canola oil in the frying treatment. Farahmandfar et al. [55] reported that major fatty acid content in canola oils is oleic acid (65%) and linoleic acid (16%).

Mackerels are known to contain high levels of DHA and EPA. In this study, DHA and EPA of raw CM were found to be 12.32% and 7.25%, respectively. After frying, both DHA and EPA levels decreased. DHA and EPA of CM decreased by 44.40% and 43.86%, respectively, after vacuum frying treatment, while they decreased 51.70 and 51.03%, respectively, after deep frying treatment. The high decrease in DHA and EPA in the deep-fried sample was due to the use of high temperatures during processing and oxygen exposure, which causes lipid oxidation. The result was confirmed by the higher lipid oxidation (TBARS value) seen in the deep-fried sample than in the vacuum-fried sample. DHA and EPA are categorized as polyunsaturated fatty acids (PUFAs), which are easily oxidized and produce unpleasant flavors [56]. Hence, high lipid oxidation may be one of the factors affecting consumer acceptance.

The total EPA and DHA of the final product VBFS was 1.34 g (6.98% of 19.19 g lipid/100 g product); the recommended daily intake of EPA and DHA is 0.5 g/day to reduce the risk of cardiovascular disease (CVD), and 1 g/day to treat any existing CVD [57]. In addition to EPA and DHA, other lipid indices were also used to assess lipid quality, including hypocholesterolemic/hypercholesterolemic (h/H), atherogenic (AI), and thrombogenic (TI) indices, and ratios of ω6 and ω3 (ω6/ω3). CVD risk is related to the type of lipid rather than the total amount of lipids in food. The atherogenic (AI) and thrombogenic (TI) indices of foods are related to coronary heart disease [58]. The h/H index is a ratio of hypocholesterolemic to hypercholesterolemic indices, which indicates the effect of specific lipids on cholesterol metabolism [59]. A higher value of h/H is required for the good health of humans. In this study, the h/H index for raw CM was 2.1, and that for processed CM was 3.53–7.45. The highest h/H index was obtained from the vacuum-fried sample. The increase in the h/H index was related to the decrease in SFA and the increase in MUFA in the processed CM. This explained why vacuum frying provided improved batter-fried CM that could be considered a healthier food.

The AI and TI are indices that describe the ratio between the main saturated fatty acids as pro-atherogenic and pro-thrombogenic with the unsaturated fatty acid as an anti-atherogenic and anti-thrombogenic [59]. Lower values of both AI and TI are considered healthy conditions for preventing CVD. The AI and TI values of raw CM were 0.57 and 0.33, respectively. The values were lower in processed CM, particularly being the lowest in VBF (0.17 and 0.16, respectively). DBF showed a similar TI value (0.33) but a lower AI value (0.34). The higher AI and TI values in deep frying treatment than in vacuum frying treatment were due to the higher SFA in the deep-fried samples, which caused a higher content of pro-atherogenic and pro-thrombogenic properties. The presence of pro-atherogenic supported lipid adhesion to immunological cells and the circulatory system [22].

The ω6/ω3 value should be considered in the human diet. A high ω6/ω3 ratio is known to induce CVD, cancer, inflammatory diseases, and autoimmune diseases [56]. The ω6/ω3 ratio of processed CM was higher than that of raw CM. The vacuum-fried CM provided a better ω6/ω3 ratio than the deep-fried CM. These results confirmed that the use of vacuum frying provides better nutrition than the use of deep frying. The recommended ω6/ω3 ratio in healthy food is 4–1 or lower [60]; therefore, the ω6/ω3 ratios in all samples in this study were considered ideal.

### 3.5. Shelf Life Prediction of Vacuum Batter-Fried Chub Mackerel with Sauce

Considering the promising use of vacuum fryers, batter-fried CM with sauce was evaluated for its shelf life as a ready-to-heat food. The prediction of shelf life was performed using the Arrhenius approach with three different storage temperatures for a total of 75 days (with 15-day intervals). The data for OA, VBN, TBARS, and TBC were obtained and are shown in Table 10. The evaluation of VBN, TBARS, and TBC for the shelf life prediction was related to microbial growth and lipid oxidation. Changes in these parameters would consequently affect consumer acceptance [49]. Thus, OA data were also included in the analysis. Each series of data was analyzed using the Visual Shelf Life Simulator from the MFDS. Changes in the final product were modeled using zero- or first-order kinetic reactions [61]. The determination of the model (zero- or first-order) was based on the largest coefficient of determination (*R*^2^) in each dataset.

As shown in Table 10, the OA data decreased with increasing storage time; this indicated a decrease in quality based on consumer acceptance. The OA data for 75 days of storage at three different temperatures ranged around 8.25–8.92, which can be considered as “like very much”. The predicted shelf life based on OA data was obtained using the Arrhenius equation for zero-order reaction kinetics. The linear regression of zero-order followed y = −7.33 + 672.81x and was used to calculate the shelf life at −18 °C storage. The rejection criterion of OA for the shelf-life prediction corresponded to a score of 5, which was the threshold in sensory analysis. As per the data, the vacuum batter-fried CM with sauce may be stored for up to 14 months.

The VBN and TBARS values increased beyond 75 days of storage. The VBN values were higher at lower freezing temperatures. At a temperature of −13 °C, VBN values reached 13.11 mg%, whereas at −18 and −23 °C, the values were 12.29 and 12.17 mg%, respectively. The linear regression of the Arrhenius equation at zero-order reaction kinetics followed y = 10.93 − 3829.02x. Using this equation, the shelf life at −18 °C storage, with a rejection criterion of 25 mg%, was 27 months. The TBARS values also showed a similar trend of increase during the 75 days of storage at three different temperatures. The highest lipid oxidation occurred at −13 °C after 75 days of storage. However, the increase in VBN and TBARS values was not significant during the 75 days of storage. Linear regression of the TBARS data was obtained from a zero-order kinetic reaction. The obtained equation was y = 1.19 − 1452.53x, and based on the rejection criterion of 5 mg MDA/kg, the product shelf life was 11 months.

The prediction of shelf life was also performed by evaluating its biological safety. Pathogenic bacteria, such as *Salmonella*, *Staphylococcus aureus*, and coliform groups, were not detected during the 75 days of storage. The TBC increased with the increase in both storage time and temperature, ranging from 2.19–2.86 log CFU/g. Shelf life predicted based on the TBC data, using the Arrhenius equation y = 1.11 − 1579.45x, was 14 months, with a rejection criterion of 5 log CFU/g. To summarize the shelf life of the final product based on OA, VBN, TBARS, and TBC, the shortest shelf life was considered in the prediction. The estimated shelf life was corrected by multiplying the Arrhenius calculation result with a safety factor of 0.8 [23], considering the temperature fluctuations during distribution in the market. Overall, we found that the vacuum batter-fried mackerel with sauce can be stored in the market for nine months.

## 4. Conclusions

Chub mackerel (CM) is a rich source of proteins and lipids, especially DHA and EPA. Conventional frying methods, such as deep frying, have been shown to reduce the quality of CM. The production of batter-fried CM with sauce using vacuum frying provided better results regarding the preservation of nutritional composition and sensory, chemical, and microbiological properties. Vacuum frying, optimized with RSM at 95 °C for 7 min 42 s, produced higher protein, lipid, DHA, and EPA levels, as well as better sensory properties than deep frying. Lipid atherogenic (AI), thrombogenic (TI), and h/H indices of the vacuum-fried product showed better values suitable for human health. The addition of sauce to the vacuum batter-fried CM (VBFS) increased the sensory properties and provided good nutritional values for human consumption. Therefore, VBFS, as a ready-to-heat product, is safe and acceptable to consumers for up to 9 months of storage at −18 °C.

## Figures and Tables

**Table 1:** The composition of the sauce used for preparing braised mackerel with radish.

Ingredients	(%)
Corn syrup	40.0
Sugar	6.0
Tomato ketchup	10.0
Chili powder	1.2
Spicy chili powder	2.5
Chopped garlic	4.0
Purified water	3.5
Soy sauce	7.0
Apple juice	3.0
Oligosaccharide	12.0
Starch	0.2
Oyster sauce	3.0
Apple cider vinegar	1.0
Mayonnaise	0.7
Brewed vinegar	1.0
Cooking wine	1.8
Lemon juice	2.0
Monosodium glutamate	0.4
Pepper powder	0.4
Citric acid	0.3

**Table 2:** Overall acceptance (OA), volatile basic nitrogen (VBN), and thiobarbituric acid reactive substances (TBARS) in vacuum batter-fried (VBF) and deep batter-fried (DBF) chub mackerel in central composite design.

Frying Method	Std. Order	Frying Condition		OA (Score)	VBN (mg%)	TBARS (mg MDA/kg)
		Temperature (°C)	Time (min)			
VBF	1	90	5.0	4.13	10.53	1.40
	2	100	5.0	6.52	11.94	1.35
	3	90	9.0	7.25	12.99	1.43
	4	100	9.0	6.03	11.58	1.52
	5	88	7.0	4.02	12.29	1.27
	6	102	7.0	5.90	11.58	1.73
	7	95	4.0	4.51	13.69	1.28
	8	95	10.0	7.25	11.23	1.74
	9	95	7.0	8.39	11.94	1.43
	10	95	7.0	8.70	11.58	1.41
	11	95	7.0	8.51	12.29	1.17
DBF	1	180	4.5	7.42	12.64	1.59
	2	200	4.5	7.26	12.99	1.40
	3	180	6.5	6.70	13.34	1.66
	4	200	6.5	6.60	12.29	1.71
	5	176	5.5	7.09	13.34	1.31
	6	204	5.5	6.98	13.69	1.76
	7	190	4.0	7.39	14.39	1.38
	8	190	7.0	7.56	12.64	1.64
	9	190	5.5	8.38	12.29	1.32
	10	190	5.5	8.20	12.99	1.57
	11	190	5.5	8.13	11.94	1.59

**Table 3:** The probability value (*p*-value) of response surface regression (ANOVA) for overall acceptance (OA), volatile basic nitrogen (VBN), and thiobarbituric acid reactive substances (TBARS) in vacuum batter-fried (VBF) and deep batter-fried (DBF) chub mackerel.

Method	Variable	*p*-Value			
		Model	Linear	Square	2-Way Interaction
VBF	OA	0.001	0.000	0.000	0.008
	VBN	0.707	0.485	0.846	0.200
	TBARS	0.311	0.591	0.509	0.673
DBF	OA	0.037	0.009	0.023	0.251
	VBN	0.109	0.149	0.076	0.110
	TBARS	0.930	0.814	0.975	0.649

**Table 4:** The predictive response surface model of overall acceptance (OA) of vacuum batter-fried (VBF) and deep batter-fried (DBF) chub mackerel at different heating temperatures (X_1_) and times (X_2_).

Response	Predictive Model	*R* ^2^	Lack of Fit (*p*-Value)
VBF	−693.3 + 13.71X_1_ + 12.79X_2_ − 0.06831X_1_^2^ − 0.2731X_2_^2^ − 0.0903X_1_X_2_	97.00%	0.081
DBF	−250.6 + 2.604X_1_ + 4.37X_2_ − 0.00687X_1_^2^ − 0.394X_2_^2^	80.55%	0.539

**Table 5:** Sensory properties of vacuum batter-fried (VBF), vacuum batter-fried and sauced (VBFS), deep batter-fried (DBF), and deep batter-fried and sauced (DBFS) chub mackerel.

Sensory Property	VBF	VBFS	DBF	DBFS
Appearance	8.68 ± 0.18 ^a^	9.00 ± 0.00 ^a^	8.20 ± 0.20 ^b^	9.00 ± 0.00 ^a^
Aroma	8.63 ± 0.19 ^ab^	8.77 ± 0.15 ^a^	8.17 ± 0.15 ^b^	8.73 ± 0.12 ^a^
Texture	8.52 ± 0.45 ^a^	8.80 ± 0.12 ^a^	8.48 ± 0.08 ^a^	8.67 ± 0.09 ^a^
Taste	8.70 ± 0.20 ^a^	8.90 ± 0.10 ^a^	8.15 ± 0.13 ^b^	8.73 ± 0.15 ^a^
Overall Acceptance	8.63 ± 0.17 ^a^	8.92 ± 0.08 ^a^	8.25 ± 0.03 ^b^	8.85 ± 0.03 ^a^

Notes: Scores are expressed as the mean ± standard error of mean. The means of each sensory property with different letters are significantly different according to Tukey’s test (*p* < 0.05).

**Table 6:** Chemical (VBN and TBARS) and microbial (TBC) properties of raw, vacuum batter-fried (VBF), vacuum batter-fried and sauced (VBFS), deep batter-fried (DBF), and deep batter-fried and sauced (DBFS) chub mackerel.

Parameters	Raw	VBF	VBFS	DBF	DBFS
VBN (mg%)	7.84 ± 0.42 ^a^	11.94 ± 0.20 ^b^	11.13 ± 0.48 ^b^	12.40 ± 0.31 ^b^	11.74 ± 0.17 ^b^
TBARS (mg MDA/kg)	1.02 ± 0.29 ^a^	1.34 ± 0.08 ^b^	1.28 ± 0.04 ^ab^	1.49 ± 0.09 ^b^	1.38 ± 0.02 ^b^
TBC (log CFU/g)	3.20 ± 0.02 ^a^	2.72 ± 0.10 ^b^	2.19 ± 0.05 ^c^	2.77 ± 0.05 ^b^	2.14 ± 0.07 ^c^

Notes: Values are expressed as the mean ± standard error of mean. The means of each parameter with different letters are significantly different according to Tukey’s test (*p* < 0.05).

**Table 7:** The nutritional composition of raw, vacuum batter-fried (VBF), vacuum batter-fried and sauced (VBFS), deep batter-fried (DBF), and deep batter-fried and sauced (DBFS) chub mackerel (per 100 g).

Composition	Raw	VBF	VBFS	DBF	DBFS
Calories (kcal)	196.99	280.67	355.86	265.48	348.14
Sodium (mg)	67.99	0.23	318.64	0.17	333.31
Carbohydrate (g)	0.03	7.01	31.52	6.26	31.04
Sugars (g)	0.00	0.06	21.87	0.00	20.50
Dietary fiber (g)	1.96	5.89	1.49	4.84	1.68
Crude lipid (g)	13.59	28.09	19.19	21.88	21.40
Crude protein (g)	15.44	36.40	17.53	27.35	12.59
Iron (mg)	1.15	2.65	1.04	1.16	0.91
Potassium (g)	0.36	0.55	0.23	0.26	0.16
Calcium (g)	0.09	0.12	0.06	0.11	0.05
Moisture (%)	69.36	24.02	40.96	39.54	53.43

**Table 8:** The amino acid profile of raw, vacuum batter-fried (VBF), vacuum batter-fried and sauced (VBFS), deep batter-fried (DBF), and deep batter-fried and sauced (DBFS) chub mackerel (g/100 g).

Amino Acid	Raw	VBF	VBFS	DBF	DBFS
Essential	4.29	16.44	7.27	8.09	3.55
Threonine	0.59	1.63	0.82	0.83	0.40
Valine	0.65	2.02	0.76	0.99	0.38
Isoleucine	0.44	1.69	0.65	0.82	0.31
Leucine	0.80	2.97	1.41	1.46	0.71
Phenylalanine	0.50	1.45	0.67	0.73	0.36
Lysine	0.86	3.37	1.56	1.64	0.71
Methionine	0.12	1.35	0.57	0.58	0.26
Tryptophan	0.14	0.24	0.15	0.15	0.07
Histidine	0.19	1.72	0.68	0.89	0.35
Non-essential	6.15	19.48	9.74	9.99	5.27
Arginine	0.55	2.15	1.02	1.06	0.50
Aspartic acid	1.12	3.48	1.69	1.74	0.81
Serine	0.65	1.37	0.77	0.71	0.40
Glutamic acid	1.40	5.33	2.83	2.79	1.68
Proline	0.56	1.55	0.76	0.89	0.49
Glycine	0.64	2.02	0.90	1.03	0.50
Alanine	0.62	2.27	1.10	1.12	0.55
Tyrosine	0.25	0.82	0.44	0.40	0.21
Cystine	0.36	0.49	0.23	0.25	0.13
Total	10.44	35.92	17.01	18.08	8.82

**Table 9:** The lipid profile and indices of raw, vacuum batter-fried (VBF), vacuum batter-fried and sauced (VBFS), deep batter-fried (DBF), and deep batter-fried and sauced (DBFS) chub mackerel (% of total lipids).

Item	Raw	VBF	VBFS	DBF	DBFS
Lauric acid (%)	0.06	0.02	0.03	0.03	0.02
Myristic acid (%)	4.31	1.33	2.16	2.33	1.45
Pentadecanoic acid (%)	0.54	0.21	0.32	0.36	0.20
Palmitic acid (%)	20.44	9.31	15.59	15.87	18.50
Magaric acid (%)	0.63	0.29	0.34	0.50	0.32
Stearic acid (%)	5.67	3.02	2.85	4.89	4.16
Arachidic acid (%)	0.43	0.58	0.26	0.49	0.21
Heneicosylic acid (%)	0.21	0.08	nd	0.13	nd
Behenic acid (%)	nd	nd	0.14	nd	0.11
Lignoceric acid (%)	1.68	0.48	0.31	0.83	0.49
Myristoleic acid (%)	0.22	0.07	0.04	0.12	0.03
Pentadecenoic acid (%)	0.11	0.04	0.10	0.06	0.11
Palmitoleic acid (%)	3.36	1.19	1.62	1.93	1.12
Magaoleic acid (%)	0.6	0.23	0.13	0.49	0.24
Oleic acid (%)	22.94	30.73	27.72	25.21	23.77
Linoleic acid (%)	5.22	31.20	32.64	30.07	36.09
γ-Linolenic acid (%)	0.1	0.05	nd	0.05	nd
Linolenic acid (%)	3.22	6.09	5.22	3.36	3.72
Eicosenoic acid (%)	4.57	1.98	2.11	1.45	1.78
Eicosadienoic acid (%)	0.28	0.13	0.04	0.14	0.08
Arachidonic acid (%)	0.94	0.31	0.20	0.59	0.35
Erucic acid (%)	4.34	1.47	0.71	1.28	0.58
Nervonic acid (%)	0.57	0.27	0.16	0.32	0.18
Docosapentaenoic acid (%)	nd	nd	0.33	nd	0.30
DHA (%)	12.32	6.85	4.75	5.95	4.29
EPA (%)	7.25	4.07	2.23	3.55	1.90
∑SFA (%)	33.97	15.32	22.00	25.43	25.46
∑PUFA (%)	24.33	31.77	30.45	43.71	46.73
∑MUFA (%)	41.71	52.91	47.55	30.86	27.81
∑ω3 (%)	20.79	17.01	12.53	12.86	10.21
∑ω6 (%)	3.54	14.76	17.92	30.85	36.52
ω6/ω3	0.17	0.87	1.43	2.40	3.58
TI	0.33	0.16	0.29	0.33	0.38
AI	0.57	0.17	0.31	0.34	0.33
h/H	2.10	7.45	4.12	3.78	3.53

Notes: ∑SFA, saturated fatty acid; ∑PUFA, polyunsaturated fatty acid; ∑MUFA, monounsaturated fatty acid; DHA, docosahexaenoic acid; EPA, eicosapentaenoic acid; AI, atherogenic index; TI, thrombogenic index; h/H, hypocholesterolemic index/hypercholesterolemic index.

**Table 10:** OA, VBN, TBARS, and TBC of vacuum batter-fried chub mackerel with sauce during storage at three different freezer temperatures for 75 days ^a^.

Temperature (°C)	Day	OA (Score)	VBN (mg%)	TBARS (mg MDA/kg)	TBC (log CFU/g)
−13	0	8.92 ± 0.08 ^a^	11.13 ± 0.47 ^a^	1.28 ± 0.05 ^a^	2.19 ± 0.05^a^
	15	8.83 ± 0.04 ^ab^	11.58 ± 0.20 ^a^	1.41 ± 0.12 ^a^	2.69 ± 0.01 ^b^
	30	8.68 ± 0.05 ^abc^	11.82 ± 1.22 ^a^	1.68 ± 0.45 ^a^	2.73 ± 0.03 ^b^
	45	8.50 ± 0.03 ^abc^	12.05 ± 0.96 ^a^	1.83 ± 0.31 ^a^	2.76 ± 0.02 ^b^
	60	8.44 ± 0.00 ^bc^	12.40 ± 0.47 ^a^	2.08 ± 0.56 ^a^	2.78 ± 0.03 ^b^
	75	8.27 ± 0.06 ^c^	13.11 ± 0.62 ^a^	2.13 ± 0.21 ^a^	2.85 ± 0.02 ^b^
−18	0	8.92 ± 0.08 ^a^	11.13 ± 0.47 ^a^	1.28 ± 0.05 ^a^	2.19 ± 0.05 ^a^
	15	8.95 ± 0.04 ^a^	11.59 ± 0.20 ^a^	1.47 ± 0.28 ^a^	2.81 ± 0.02 ^b^
	30	8.63 ± 0.10 ^abc^	11.70 ± 0.12 ^a^	1.64 ± 0.27 ^a^	2.74 ± 0.01 ^b^
	45	8.67 ± 0.06 ^abc^	11.82 ± 0.77 ^a^	1.81 ± 0.26 ^a^	2.76 ± 0.04 ^b^
	60	8.39 ± 0.08 ^bc^	12.17 ± 0.23 ^a^	2.03 ± 0.21 ^a^	2.81 ± 0.03 ^b^
	75	8.30 ± 0.05 ^c^	12.29 ± 0.35 ^a^	2.08 ± 0.10 ^a^	2.86 ± 0.03 ^b^
−23	0	8.92 ± 0.08 ^a^	11.13 ± 0.47 ^a^	1.28 ± 0.05 ^a^	2.19 ± 0.05 ^a^
	15	8.83 ± 0.04 ^ab^	11.59 ± 0.41 ^a^	1.47 ± 0.48 ^a^	2.77 ± 0.04 ^b^
	30	8.67 ± 0.08 ^abc^	11.94 ± 0.20 ^a^	1.69 ± 0.09 ^a^	2.75 ± 0.03 ^b^
	45	8.54 ± 0.24 ^abc^	11.94 ± 0.20 ^a^	1.81 ± 0.07 ^a^	2.71 ± 0.11 ^b^
	60	8.29 ± 0.08 ^c^	12.05 ± 0.59 ^a^	1.97 ± 0.32 ^a^	2.75 ± 0.04 ^b^
	75	8.25 ± 0.07 ^c^	12.17 ± 0.31 ^a^	1.97 ± 0.14 ^a^	2.77 ± 0.05 ^b^

Notes: Values are expressed as the mean ± standard error of mean. The means of each parameters with different letters are significantly different according to Tukey’s test (*p* < 0.05).

## Data Availability

Data supporting reported results are available upon request.
